# CBX4-dependent regulation of HDAC3 nuclear translocation reduces Bmp2-induced osteoblastic differentiation and calcification in adamantinomatous craniopharyngioma

**DOI:** 10.1186/s12964-021-00797-w

**Published:** 2022-01-03

**Authors:** Xiaorong Yan, Dezhi Kang, Yuanxiang Lin, Songtao Qi, Changzhen Jiang

**Affiliations:** 1 Department of Neurosurgery, The First Affiliated Hospital of Fujian Medical University, 20# Chazhong Road, Fuzhou City, Fujian Province, China; 2 Department of Neurosurgery, Guangdong Province, Nanfang Hospital, Southern Medical University, 1838#Guang Zhou Road 1838#, Guangzhou City, 510515 China

**Keywords:** Adamantinomatous craniopharyngioma calcification, Osteoblastic differentiation, HDAC3, CBX4, Bmp2

## Abstract

**Background:**

Calcification of adamantinomatous craniopharyngioma (ACP) often causes problems with tumor resection, leading to a high incidence of deadly complications and tumor recurrence. Histone acetyltransferase (HAT) and histone deacetylase (HDAC) are 2 key enzymes that regulate histone acetylation and play important roles in tumor development. However, the roles of HAT and HDAC in the calcification and osteoblastic differentiation of ACP are not known.

**Methods:**

In this study, primary cells were isolated from ACP tissues, and calcification was induced with bone morphogenetic protein 2 (Bmp2). HDAC3 expression was assessed in 12 tissue samples by Western blotting and immunohistochemistry. ACP calcification was assessed by Alizarin red staining. A luciferase reporter assay was performed to examine the interaction between miR-181b and the 3’-untranslated region of the polycomb chromobox 4 (CBX4) gene.

**Results:**

Our results showed that the expression of HDAC3 was increased in the calcified ACP samples, but inhibition of HDAC3 promoted ACP cell calcification and osteoblastic differentiation. Mechanistically, HDAC3 nuclear translocation was suppressed by Bmp2, leading to Runx2 protein expression and Osterix, osteocalcin (OCN), osteopontin (OPN), and alkaline phosphatase (ALP) mRNA expression. In addition, this process was suppressed by CBX4, which stabilized the nuclear localization of HDAC3. miR-181b, the expression of which was increased in Bmp2-induced ACP cells, directly targeted and decreased CBX4 expression and inhibited the nuclear localization of HDAC3.

**Conclusions:**

Our results demonstrate that Bmp2 increases miR-181b levels to directly target and inhibit CBX4 expression, leading to a reduction in the CBX4-dependent regulation of HDAC3 nuclear translocation, which results in Runx2 activation/osteoblastic differentiation and calcium deposition in ACP. Further studies targeting these cascades may contribute to therapeutic interventions used for recurrent ACP.

**Video Abstract**

**Supplementary Information:**

The online version contains supplementary material available at 10.1186/s12964-021-00797-w.

## Background

**Fig. 1:**
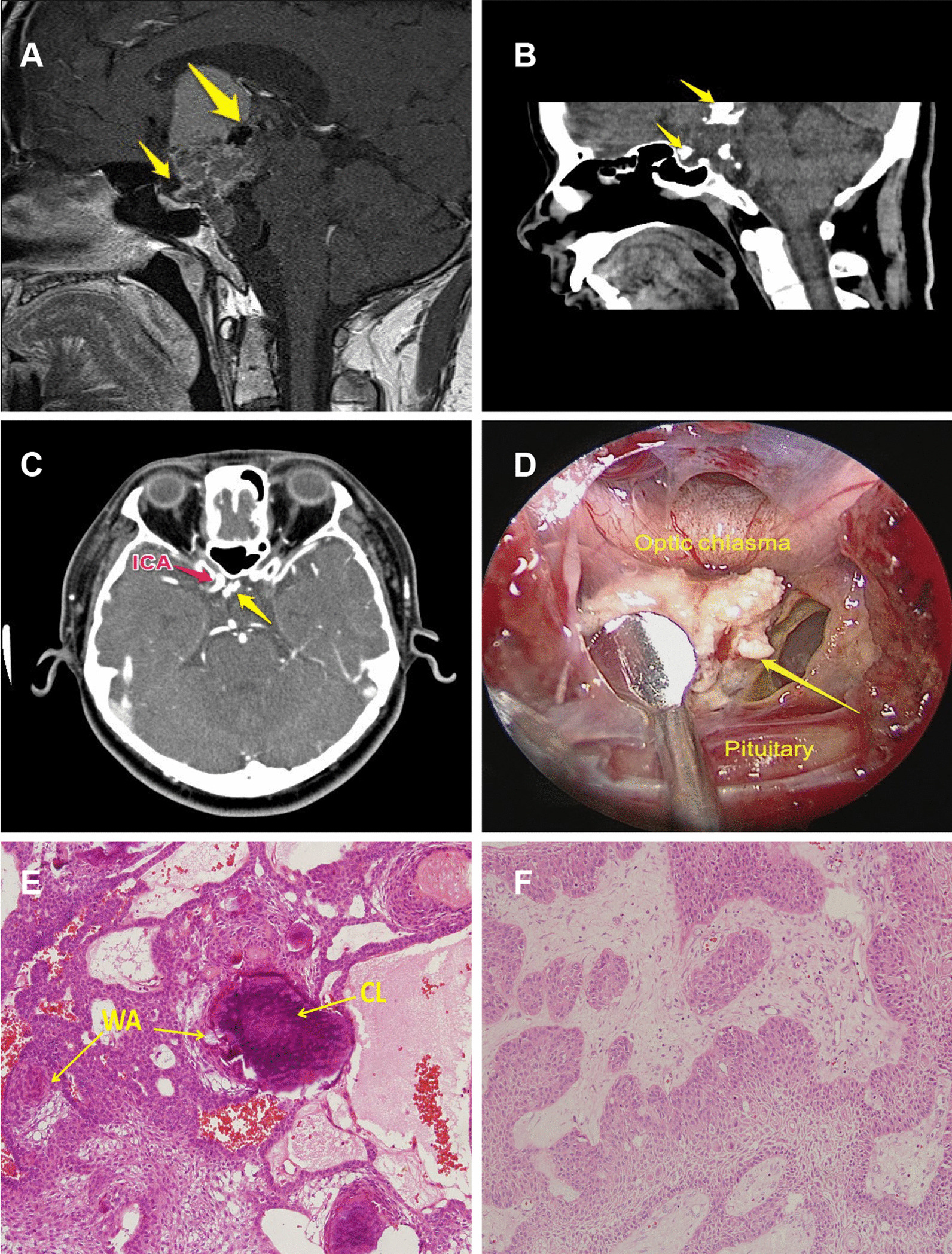
Relationship between calcification of ACP and its surrounding anatomic structures. **A**, **B** Sagittal MR and CT scans showing tumor calcifications (yellow arrows: calcification lesions are shown by the low signal in the MR image presented in **A** and high density in the CT scan presented in **B**) located above the pituitary gland and below the hypothalamus. **C** The calcification lesion (yellow arrow) was closely related to the right ICA. **D** Under the surgical view from the extended endonasal endoscopic perspective, the calcification lesion (yellow arrow) was found to adhere to the optic chiasm and pituitary gland. Surgeons must be very careful when separating the lesion from the surrounding anatomical structures (hypothalamus, ICA and optic chiasm) to avoid serious postoperative complications. (ICA: internal carotid artery). **E** Calcification lesion (CL) and its precursor structure the whorl-like array cells (WA) were found in adamantinomatous craniopharyngioma sample. We can see a calcification lesion (CL) is arising from its whorl-like array cells (WA) at the central part of this image. **F** Squamous papillary craniopharyngioma comprising mature squamous epithelia without calcification

Craniopharyngioma (CP) is a tumor that typically arises in the sellar/suprasellar region and accounts for 4.7–7.9% of all primary intracranial neoplasms [1]. CP is among the most common calcified tumors in the central nervous system [2]. Considering the importance of tumor-adjacent structures, such as the hypothalamus, internal carotid artery, optic nerve and pituitary stalk, calcification often causes problems during tumor resection, leading to a high incidence of deadly complications and tumor recurrence (Fig. 1A-D) [1, 2]. CP calcification mostly occurs in adamantinomatous craniopharyngioma (ACP), while calcification is rarely seen in squamous papillary craniopharyngioma (SPCP) [3]. Histologically, cell pattern of ACP is different from SPCP (Fig. 1E, F). This difference may be explained by the theory that ACP originates from odontogenic rests associated with remnants of Rathke’s pouch, while SPCP develops from buccal mucosa rests [3, 4].

In our previous study, we showed that ACP calcification can be considered a consequence of cell differentiation and might resemble the calcium deposition seen in osteogenesis and odontogenesis [3]. Expression of the core marker of osteogenic differentiation, Runt-related transcription factor 2 (Runx2), is high in severely calcified CPs. Runx2 is a transcription factor that regulates downstream factors associated with calcium deposition during osteogenesis/odontogenesis [5, 6]. In our previous work, we found that Bmp2-induced osteogenic differentiation and activation of Runx2 signaling play important roles in ACP calcification [3].

Histone acetyltransferase (HAT) and histone deacetylase (HDAC) are 2 key enzymes that regulate histone acetylation [7–9]. HAT transfers the acetyl group of acetyl-CoA to specific lysine residues at the amino terminus of histones to loosen nucleosomes, expose DNA, and promote mRNA transcription [10, 11]. HDAC removes acetyl groups from histones, promoting compaction of DNA and histones, which inhibits mRNA transcription [12]. Choung et al. [13] reported that HDAC3 inhibits Smad4 activity and thus reduces downstream mRNA transcription in human dental pulp stem cells. However, it is unknown whether HAT and/or HDAC are expressed in ACP cells and whether either plays a role in the regulation of the nuclear transcription factor Runx2.

In this study, we provide evidence showing that HDAC3 reduces osteoblastic differentiation and calcification in ACP cells treated with bone morphogenetic protein 2 (Bmp2) by suppressing Runx2 signaling pathways. Moreover, CBX4 stabilizes the nuclear localization of HDAC3 to inhibit Runx2 expression.

## Methods

### Primary culture of ACP cells

6 patients with primary ACP who underwent surgery from August 2017 to February 2020 at the First Affiliated Hospital of Fujian Medical University were enrolled in this stage. 2 male and 4 female patients were enrolled and the average age of the patients at the time of surgery was 43.33 ± 15.45 years old (range 19–65 years). All patients and/or their legal surrogates provided written informed consent for the use of the tissue specimens. The study was approved by the Ethics Committee of the First Affiliated Hospital of Fujian Medical University. Primary ACP cells were cultured and identified as previously reported [14]. Briefly, after tumor resection, solid tumor samples were immediately placed into a cube with DMEM medium (Gibco, Grand Island, USA) containing 10% (v/v)fetal calf serum (Life Technologies, Basel, Switzerland) and penicillin/streptomycin. Then the specimen was delivered for primary cell culture. At this stage, the tumors were cut into small pieces of 1 mm diameter and dispersed by treatment with trypsin for 30 min at 37 °C. Specimens were then filtered and the cell suspension was centrifuged at 800 rpm for 5 min. The cells were washed and then cultured at 37 °C in 5% CO2 atmosphere in a keratinocyte medium (Gibco, Grand Island, USA) with 2 × 105 cells/ml. The cells were characterized by immunocytochemistry. Staining for cytokeratin ((CK, dilution 1:100; 85 ZM-0069, ZSGB-BIO, Beijing, China).

### In vitro calcification assay

ACP cells were cultured with Bmp2 for 10 days and then stained with 2% Alizarin red (Sigma-Aldrich). Briefly, after the medium was removed, ACP cells were rinsed with PBS and fixed with 4% paraformaldehyde for 30 min at room temperature. Next, the cells were rinsed twice with PBS and stained with a 2% alizarin red (ph 4.2) (Sigma-Aldrich, St. Louis, MO, USA) working solution for 10 min at room temperature. Finally, the cells were washed with PBS three times, and images were collected.

### Western blot analysis

Protein was extracted from the fresh ACP surgical specimens by lysing cells with protease inhibitor cocktail (Roche, USA). After measuring the protein content with a Bradford assay, 20 μg of protein was resolved by 10% SDS-PAGE and transferred to PVDF membranes. Membranes were blocked with 5% skim milk in TBST for 2 h at room temperature and incubated overnight with the following antibodies: anti-HAT, anti-HDAC1, anti-HDAC2, anti-HDAC3, anti-HDAC8 (1:1000, Abcam, USA), anti-CBX4, anti-Runx2, anti-histone (1:1000, Cell Signaling Technology, USA), and anti-β-actin (1:1000, Proteintech, USA). The specimens were then incubated with secondary antibodies, IRDye800-conjugated anti-rabbit IgG and IRDye680-conjugated anti-mouse IgG (1:15,000, LiCor, USA), for 1 h at room temperature to label the primary antibody. An Odyssey Infrared Image System (LiCor, USA) was used to analyze signal intensities. The densitometry results were first normalized to the density obtained for β-actin or histone and then compared with that of the control to obtain relative fold changes.

### Immunohistochemistry (IHC)

Paraffin-embedded tissue sections from 12 patients with pathologically confirmed ACP were obtained from the pathology department of the First Affiliated Hospital of Fujian Medical University. 5 male and 7 female patients were enrolled and the average age of the patients at the time of surgery was 46.42 ± 16.02 years old (range 14–65 years). These patients underwent surgery from July 2017 to February 2020. Eight of the patients had been confirmed to have obvious tumor calcification while the rest of four patients with no tumor calcification by computed tomography (CT) scan, as described in previous reported [15]. The paraffin sections were stained with anti-HDAC3 antibody (1:100, Abcam, USA) as previously reported [16].

### miRNA, siRNA, and vector construct transfection

siRNA targeting HDAC3 for knockdown, miR-129-5p, miR-144, miR-181b, miR-181c, miR-195, miR-200b, miR-410 mimics (mimic-129-5p, mimic-144, mimic-181b, mimic-181c, mimic-195, mimic-200b, and mimic-410), miR-181b inhibitor (inh-181b), miR-200b inhibitor (inh-200b), miR-410 inhibitor (inh-410), siHDAC3, si-CBX4 and negative control (NC) oligonucleotides were purchased from RiboBio (Guangzhou, People’s Republic of China) and transfected into cells using Lipofectamine 3000 (Invitrogen, USA) at a concentration of 50 nM. Q-PCR and western blot was used to assay the transfection efficiency. CBX4-expressing vectors with miR-181b binding sites or mutated miR-181b seed sequences in the MCU 3’-untranslated region (MUT) were purchased from Cyagen Biosciences Inc. (Guangzhou, China). The vectors were subcloned into a psiCHECK-2 vector and then transfected into cells using Lipofectamine 3000 (Invitrogen, USA).

### Luciferase assays

The, 3’-UTR of CBX4 and the mutated 3’-UTR of CBX4 were amplified and inserted downstream from the stop codon of Renilla luciferase using a psiCHECK-2 vector (Sagene, China). HeLa cells were cultured in 96-well plates and cotransfected with 10 ng of psiCHECK-2-MCU and 5 pmol of mimic-181b or NC. After incubation for 48 h, firefly and Renilla luciferase activity levels were measured using a dual-luciferase reporter assay system (Promega, Madison, WI) [17].

### Immunofluorescence staining

Cells were cultured on a confocal petri dish (NEST Biotechnology Co. Ltd. China), fixed with 4% formaldehyde, permeabilized with 0.1% Triton X-100, and blocked with 5% bovine serum albumin (BSA) for 30 min at room temperature. The cells were stained with anti-HDAC3 antibody (1:100, Abcam, USA), which had been diluted 1:200 in 5% goat serum, overnight at 4 °C. The cells were subsequently stained with Alexa Fluor 594 (R37119) (1:200 dilution in PBS) (Invitrogen) at room temperature for 1 h, followed by incubation with DAPI (1:1000 dilution in PBS) for 5 min (min). The cells were then examined with a Lecia laser scanning microscope (FV1000, Olympus) at 100× magnification [18].

### Bimolecular fluorescence complementation (BiFC) assay

The BiFC assay was performed to further explore the interaction between CBX4 and HDAC3 in vivo according to previously reported [19]. In brief, the coding region of CBX4 was cloned into pBiFC-mCherryN159. The coding regions of HDAC3 cloned into pBiFC-mCherryC160. pBiFC-mCherryN159-CBX4 and pBiFC-mCherryC160-HDAC3 were co-transfected into ACP cells. 48 h post-transfection, the cells were incubated with Hoechst 33258 for nuclear staining and observed under the laser confocal microscope (FV1000, Olympus).

### RNA extraction and miRNA analysis

Total RNA was extracted from cells using TRIzol (Invitrogen, Carlsbad, CA). The quantity of isolated RNA was determined with a NanoDrop ND-2000 spectrophotometer (Nanodrop Technologies, Delaware, USA). Next, 1000 ng of total RNA was reverse transcribed using a TaqMan microRNA reverse transcription kit (ABI, Forest City, CA). The mRNA and miRNA levels were quantified by qRT-PCR using SYBR Green (Roche, USA) and TaqMan assay kits (ABI) with GAPDH and U6 snRNA used as references, respectively, as previously described [20, 21]. The assays were performed on a 7500 FAST instrument (ABI) under standard conditions as recommended by the manufacturer: 95 °C for 10 min, followed by 40 cycles of 95 °C for 15 s and 60 °C for 1 min. A melting curve analysis was then performed. Relative mRNA and miRNA levels were calculated according to the 2-Δcycle threshold method. The sequences of the PCR primers are shown in Table 1.

**Table 1:** List of qPCR primers for Osterix, OCN, OPN, and ALP

Gene	Primer Sequence
**Osterix**	
Forward	5′-TCCCTGGATATGACTCATCCCT-3′
Reverse	5′-CCAAGGAGTAGGTGTGTTGCC-3′
**OCN**	
Forward	5′-GGCGCTACCTGTATCAATGG-3′
Reverse	5′-GTGGTCAGCCAACTCGTCA-3′
**OPN**	
Forward	5′-GGAGTTGAATGGTGCATACAAGG-3′
Reverse	5′-CCACGGCTGTCCCAATCAG-3′
**ALP**	
Forward	5'-CCAACTCTTTTGTGCCAGAGA-3'
Reverse	5'-GGCTACATTGGTGTTGAGCTTTT-3'
**GAPDH**	
Forward	5'-CCGCATCTTCTTTTGCGTCG-3'
Reverse	5'-GGACTCCACGACGTACTCAG-3'

### Statistical analysis

Cell culture experiments were repeated a minimum of 3 times. All quantitative xenograft and in vitro assay results are presented as the means ± standard deviation. Statistical analyses were conducted using SPSS version 13.0 software. All statistical tests were 2-sided. Student’s *t*-test for bar graph was performed. *P* values < 0.05 were considered to indicate statistical significance.

## Results

### HDAC3 expression is increased in calcified ACP

**Fig. 2:**
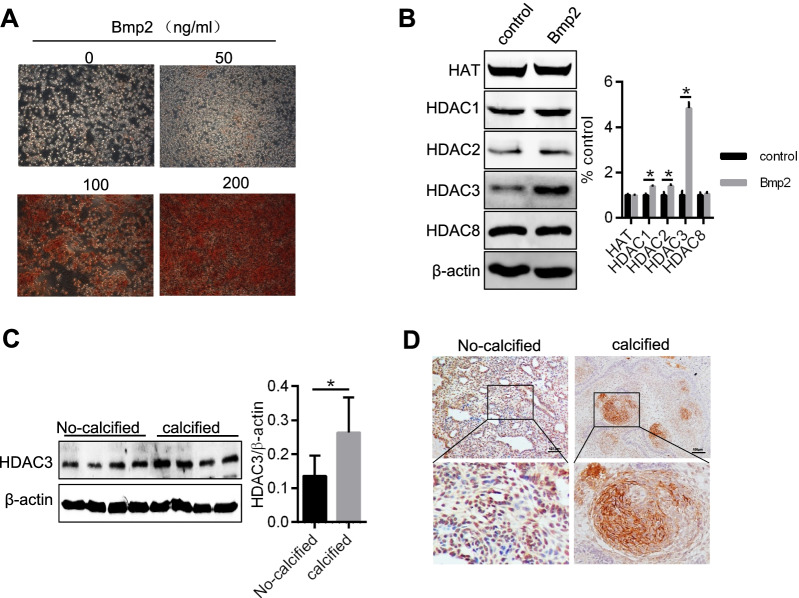
The expression of HDAC3 is increased in calcified ACP tissue. **A** ACP cells were treated with Bmp2 (0, 50, 100 and 200 ng/ml) for 10 days and then stained with 2% Alizarin red. **B** Western blotting was used to detect protein levels of HAT, HDAC1, HDAC2, HDAC3, and HDAC8 in ACP cells treated with Bmp2 (200 ng/ml) for 10 days. **C**, **D** HDAC3 protein levels were analyzed in noncalcified (n = 4) and calcified (n = 8) ACP tissue by Western blotting (left) and immunohistochemistry (right). In the IHC experiments, HDCA3 mainly showed strong cytoplasmic staining in calcified ACP samples. In contrast, in noncalcified ACP samples, the main area of HDAC3 expression was the nucleus. *P < 0.05

To explore the effects of histone acetylation with respect to ACP calcification, we measured the HAT and HDAC3 levels in Bmp2-treated ACP cells. The expression of HDAC3 was significantly increased (Fig. 2A, B). Examination of the surgical specimens showed that HDAC3 protein levels were increased in the calcified tissue compared to those in noncalcified tissue (Fig. 2 C). In the IHC studies, HDCA3 showed strong cytoplasmic staining of calcified ACP samples. In contrast, in noncalcified ACP samples, the main area of HDAC3 expression was the nucleus (Fig. 2 D).

### Bmp2 suppressed HDAC3 nuclear translocation to activate the Runx2 pathway and promote osteoblastic differentiation of ACP

**Fig. 3:**
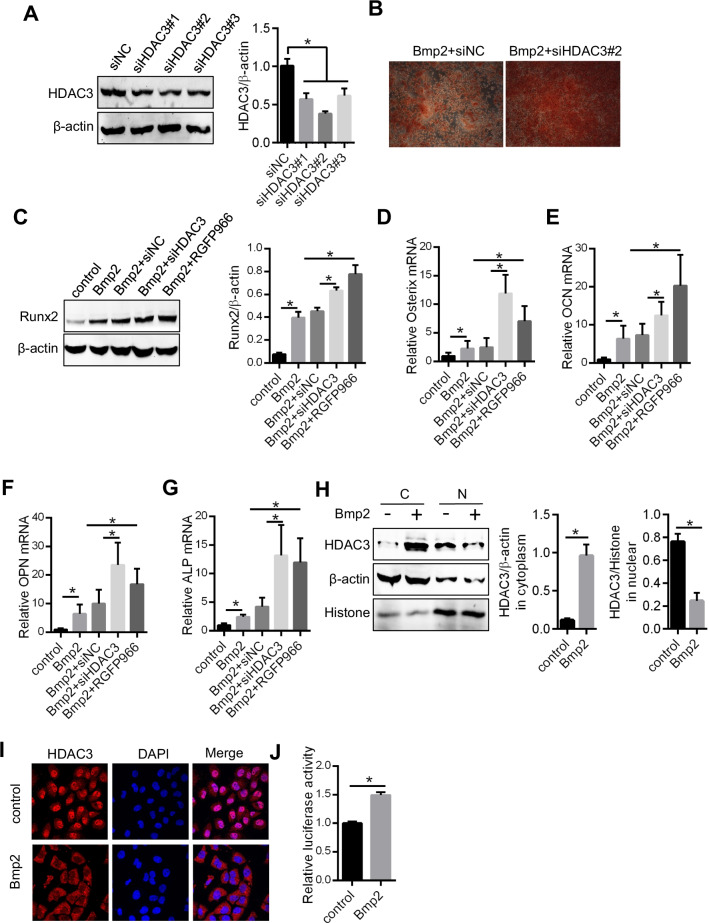
Bmp2 suppressed HDAC3 nuclear translocation to activate the Runx2 signaling pathway and promote ACP osteoblastic differentiation. **A** After HDAC3 protein levels were knocked down by siRNA, 200 ng/ml Bmp2 was used to treat ACP cells for 10 days, and the calcification was assessed by Alizarin red staining (**B**). **C** RGFP966 (10 µM), an inhibitor of HDAC3 activation, was used as pretreatment for 1 h and then Bmp2 (200 ng/ml) was added to cells with HDAC3 knocked down or expressed. Runx2 protein levels were analyzed by Western blotting. **D**–**G** The mRNA levels of Osterix, OCN, OPN, and ALP were examined by PCR. ACP cells were treated with 200 ng/ml Bmp2 for 10 days, and the nuclear translocation of HDAC3 was detected by Western blotting (**H**), immunofluorescence (**I**) and (**J**) a dual-luciferase reporter system. *P < 0.05

To examine the relationship between HDAC3 expression and ACP calcification, siRNA was used to knock-down the expression of HDAC3 (Fig. 3A). Inhibition of HDAC3 enhanced Bmp2-treated ACP cell calcification (Fig. 3B) and promoted Runx2 protein expression, a core marker of osteogenic differentiation, and the expression of Runx2 downstream genes (Osterix, osteocalcin (OCN), osteopontin (OPN), and alkaline phosphatase (ALP) mRNA) (Fig. 3C–G).

A previous study indicated that HDAC3 affects gene transcription upon localizing to the nucleus [22]. Thus, we separated nuclear and cytoplasmic proteins obtained from Bmp2-treated ACP cells and found that the HDAC3 protein level was increased in the cytoplasm but not in the nucleus (Fig. 3H–J). These results imply that high expression of HDAC3 may result in negative feedback and that Bmp2 suppresses HDAC3 nuclear translocation to enhance osteoblastic differentiation and cell calcification in ACP cells.

### CBX4 suppresses the Bmp2-activated Runx2 pathway and stabilizes the nuclear localization of HDAC3

**Fig. 4:**
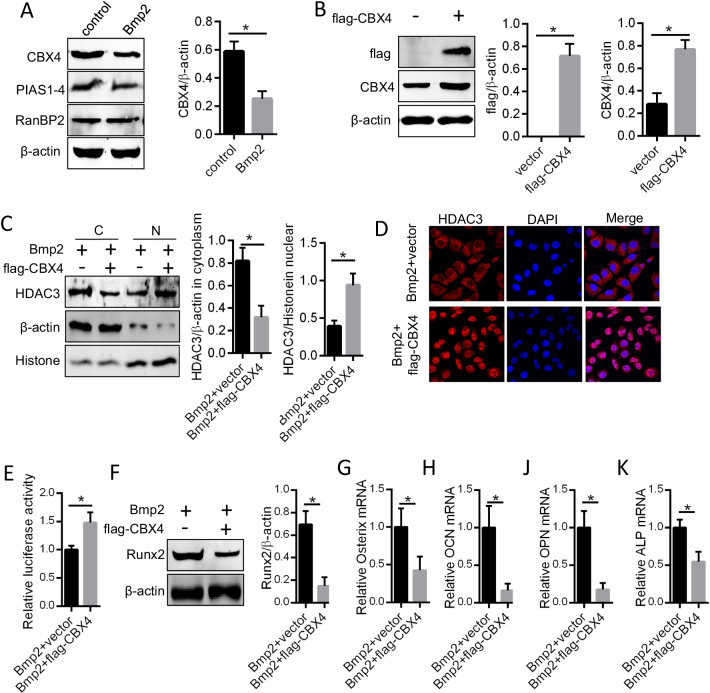
CBX4 suppressed Bmp2 activation of the Runx2 pathway by stabilizing the nuclear localization of HDAC3. **A** Small-ubiquitin-like modifier ligases CBX4, PIAS1-4, and RanBP2 in ACP cells treated with 200 ng/ml Bmp2 for 10 days were detected by Western blotting. **B** The flag-labeled CBX4 gene was transfected into ACP cells, and flag and CBX4 protein expression was quantified. ACP cells with and without flag-CBX4 over-expression were treated with Bmp2 (200 ng/ml) for 10 days, and nuclear localization of HDAC3 was detected by Western blotting (**C**), **D** immunofluorescence and **E** a dual-luciferase reporter system. **F** Western blotting was used to measure Runx2 protein levels. **G**–**K** The mRNA levels of Osterix, OCN, OPN, and ALP were determined by PCR. *P < 0.05

Small ubiquitin-like modifiers (SUMOs) are important types of protein expression modifiers and play important roles in maintaining protein stability and function [23, 24]. SUMO ligase is a key factor for small-ubiquitin-like modification [25]. We screened the 3 common small ubiquitin-like modifier ligases in Bmp2-treated ACP cells and found significantly decreased CBX4 protein expression (Fig. 4A). After over-expression of CBX4, the inhibition of HDAC3 by Bmp2 in the nucleus was reversed (Fig. 4B–E). However, the increased activation of the Runx2 pathway by Bmp2 was retained in the CBX4 over-expression group (Fig. 4F–K).On the contrary, knockdown of CBX4 resulted in decreased localization to the nuclei of HDAC3 (Additional file 1: Figure S1A–C) and activation of the Runx2 pathway (Additional file 1: Figure S1D–H). In addition, we found that there was an interaction between CBX4 and HDAC3, and that was suppressed by Bmp2 (Additional file 1: Figure S1I). These data suggest that CBX4 can stabilize the nuclear localization of HDAC3 to antagonize the effect of Bmp2 on ACP cell calcification.

### The miR-181b level is increased in Bmp2-treated ACP cells and targeted to inhibit CBX4 expression

**Fig. 5:**
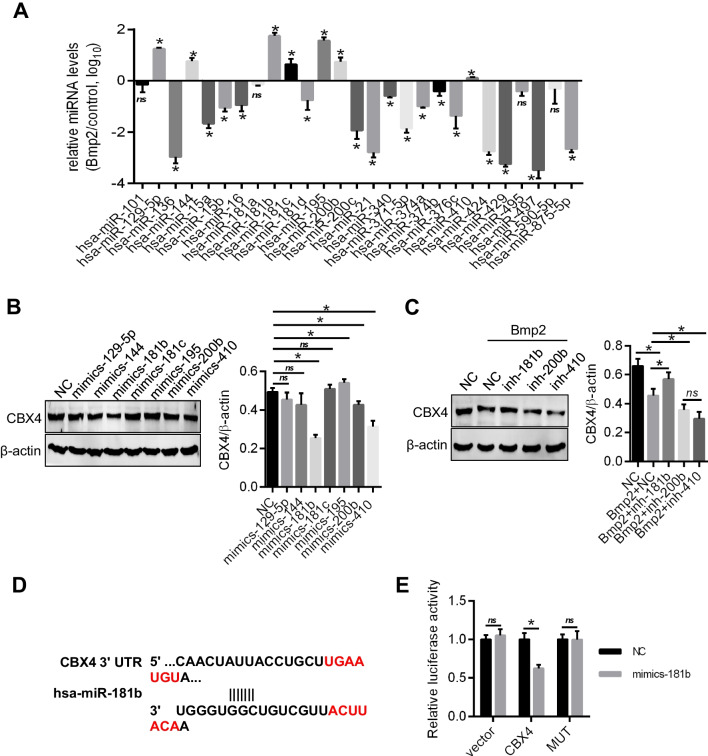
miR-181b targets CBX4 to suppress its expression. **A** PCR was performed to detect the expression levels of miRNAs which were predicted to target CBX4 by filtering the output of target prediction algorithms (TargetScan) in ACP cells with 200 ng/ml Bmp2 treatment for 10 days*vs control, *P* < 0.05, and ns = not significant. **B** The expression levels of the CBX4 protein were measured in ACP cells transfected with different miRNAs. **C** ACP cells with miRNAs knocked down by miRNA inhibitors (inh-181b, inh-200b, and inh-410) were treated with Bmp2 (200 ng/ml) for 10 days, and CBX4 protein levels were measured by Western blotting. **D** Sequences present in the 3’UTR of CBX4 targeted by miR-181b and its target region are highlighted. **E** miR-181b targeted to the segment of the 3’UTR of CBX4 decreased luciferase reporter gene activity in HeLa cells. *P < 0.05, and ns = not significant

MicroRNAs are important regulators of protein expression via suppressing protein translation or accelerating mRNA degradation [26]. By filtering the output of target prediction algorithms (TargetScan), we identified 27 miRNAs that may target CBX4 but only 7 miRNAs (miR-129-5p, miR-144, miR-181b, miR-181c, miR-195, miR-200b, and miR-410) (Fig. 5A) were significantly increased. We thus over-expressed them to tested the effect on the expression of CBX4 in ACP cells and confirmed the transfection efficacy by PCR (Figure supplement 2 A). The results showed that 3 miRNAs (miR-181b, miR-200b, and miR-410) caused a marked reduction and miR-195 induced an increase in the expression of CBX4 (Fig. 5B), but only knockdown of miR-181b blocked CBX4 low expression induced by Bmp2 (Figure supplement 2B and 5C). Therefore, miR-181b was selected for further study.

To obtain additional direct evidence that CBX4 expression is regulated by miR-181b, we predicted the binding site of miR-181b in the 3’UTR of CBX4 and found that human CBX4 was targeted by miR-181b at one region in its 3’UTR (Fig. 5D), and we performed a luciferase reporter assay. The CBX4 3’-UTR reporter or the corresponding mutant reporter (MUT) was cotransfected with mimic-181b (a miR-181b mimic) or NC (mimic controls) into 293 T cells. The results showed that compared to that in the NC, the luciferase activity of CBX4 cotransfected with mimic-181b was significantly decreased. No apparent change in luciferase activity was observed in the cells cotransfected with MUT and mimic-181b or the NC (Fig. 5E). These data indicate that CBX4 is a direct functional target of miR-181b.

### Blocking miR-181b reduces the calcification and osteoblastic differentiation of Bmp2-treated ACP cells

**Fig. 6:**
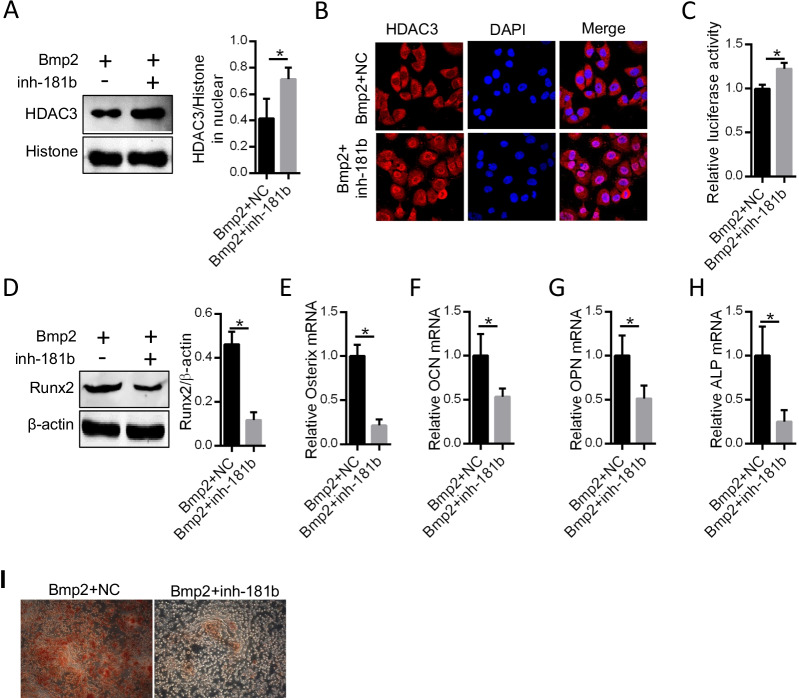
The effect of miR-181b on osteoblastic differentiation and cell calcification of ACP. After inhibition of miR-181b, ACP cells were treated with Bmp2 (200 ng/ml) for 10 days. **A** The level of HDAC3 localized to the nucleus was detected by Western blotting, and histone served as the loading control. The nuclear localization of HDAC3 was detected by **B** immunofluorescence and a dual-luciferase reporter system (**C**). **D** Western blotting was used to measure Runx2 protein levels. **E**–**H** The mRNA levels of Osterix, OCN, OPN, and ALP were measured by PCR. **I** The calcification of ACP cells was detected by Alizarin red staining. *P < 0.05

We next investigated the role of miR-181b in HDAC3 nuclear translocation and ACP cell calcification. HDAC3 nuclear translocation was increased in Bmp2-treated ACP cells subjected to miR-181b knockdown (Fig. 6A–C). However, inhibition of miR-181b decreased Runx2 protein expression, the expression of its downstream genes (Osterix, OCN, OPN, and ALP mRNA) and the ACP cell calcification degree (Fig. 6D–I). These results suggest that blocking miR-181b activity could reduce the calcification and osteoblastic differentiation of ACP cells treated with Bmp2 through loss of CBX4 inhibition.

## Discussion

Because of the important structures in the sellar region, calcification increases the difficulty of ACP resection and increases the risk of complications and recurrence [1, 2]. Our previous study showed that ACP calcification may be a result of osteogenic differentiation, which mimics the calcium deposition seen in osteogenesis and odontogenesis [3]. The activation of Runx2 signaling due to Bmp2-induced osteogenic differentiation is a key factor in this process [3]. In the present study, we demonstrated that HDAC3 reduces osteoblastic differentiation and calcification in Bmp2-treated ACP cells by suppressing Runx2 signaling pathways. Moreover, CBX4 stabilized the nuclear localization of HDAC3. In addition, increased miR-181b expression suppressed targeted inhibition of CBX4 expression in Bmp2-induced ACP cells.

Histone acetylation is one of the most extensively studied epigenetic modifications, and it plays key roles in chromatin remodeling and gene regulation and is mainly regulated by HAT and HDAC [27]. In this study, we found that HDAC3 expression was increased in calcified ACP tissue, and inhibition of HDAC3 enhanced ACP cell calcification. We further observed that HDAC3 was mainly distributed in the cytoplasm, not the nucleus, in calcified ACP tissue. Considering that HDAC regulates histone acetylation, the distribution of levels inside and outside of the nucleus may affect its function [28, 29]. Wang et al. [22] reported that HDAC3 translocation from the cytoplasm into the nucleus was critical for the proliferation and differentiation of oligodendrocyte progenitor cells. Moreover, studies have shown that HDAC can regulate the effects or expression of Runx2, which plays an important role in osteoblastic differentiation and cell calcification [30, 31]. We also found that inhibition of HDAC3 expression led to increased Runx2 expression and the expression of its downstream pro-calcification genes (Osterix, OCN, OPN, and ALP mRNA). Therefore, increased expression of HDAC3 may be a feedback mechanism triggered by the failure of its nuclear localization in calcified ACP tissue. In addition, HDAC3 may suppress ACP calcification and osteoblastic differentiation by decreasing Runx2 expression.

Small ubiquitin-like modification is an important type of protein expression modification and plays an important role in maintaining protein stability and function [23, 25, 32]. We screened 3 common SUMO E3 ligases in Bmp2-treated ACP cells and found a significant decrease in CBX4 protein expression. Recently, CBX4 has been proposed to play an important role in many cancers as an oncogenic or anti-oncogenic factor that regulates cell proliferation [33, 34], angiogenesis [23], and metastasis [35]. Our results showed that CBX4 increased the nuclear localization of HDAC3 and subsequently inhibited Runx2 signaling pathways. Inversely, knockdown of CBX4 led to decreased localization to the nuclei of HDAC3 as well as activation of the Runx2 pathway. Kang et al. [36] also reported that CBX4 maintained the nuclear localization of recruited HDAC3 in colorectal carcinoma. These data suggest that in calcified ACP cells, decreased expression of CBX4 results in loss of HDAC3 nuclear localization and inhibition of Runx2 signaling pathways.

miRNAs are short, endogenous, noncoding RNAs known to regulate the translation of target transcripts and have been implicated in many cancer processes [17, 37]. To explore whether posttranscriptional regulation by certain miRNAs is an upstream regulatory mechanism of CBX4 expression, we used several target prediction algorithms to determine miRNA binding sites in the CBX4 3′-UTR. miR-181b, miR-200b, and miR-410 were found by Western blot analysis to induce a marked reduction in the expression of CBX4, but only the knockdown of miR-181b could block Bmp2-induced low CBX4 expression. A previous study reported that miR-181b is a direct regulator of PIAS3 that activates the STAT3 signaling pathway in colon cancer [38]. The results of our study showed that miR-181b enhanced the osteoblastic differentiation and calcification of Bmp2-treated ACP cells by targeting CBX4. Taken together, these data illustrate that miR-181b can promote osteoblastic differentiation and cell calcification of ACP by targeting CBX4.

## Conclusions

**Fig. 7:**
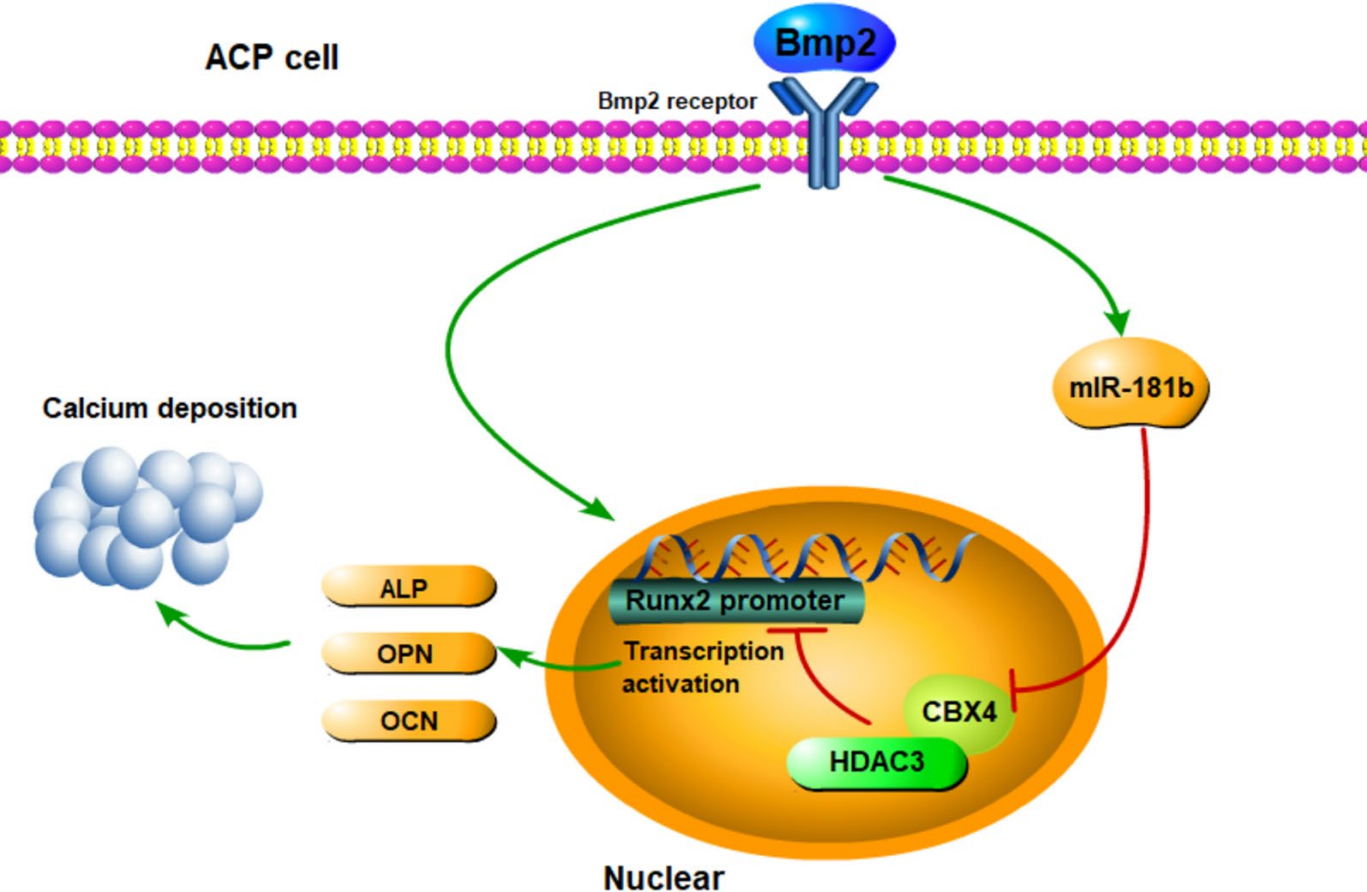
Schematic diagram of the proposed mechanism supported by our results. Bmp2 increases miR-181b levels to directly target and inhibit CBX4 expression, leading to a reduction in CBX4-dependent regulation of HDAC4 nuclear translocation. Subsequently, this process promotes Runx2 activation (osteoblastic differentiation) and calcium deposition in ACP

In conclusion, our results demonstrate that Bmp2 increases miR-181b levels to directly target and inhibit CBX4 expression, leading to a reduction in CBX4-dependent regulation of HDAC4 nuclear translocation. Subsequently, this process promotes Runx2 activation (osteoblastic differentiation) and calcium deposition in ACP (Fig. 7). These results suggest that maintaining the nuclear localization of HDAC3 may be a novel method for preventing calcification in ACP. Further exploration of the mechanisms by which CBX4 modulates HDAC3 is warranted and may contribute to the development of therapeutic interventions for ACP in the future.

## Supplementary Information

**Additional file 1: Figure S1.** Knockdown of CBX4 decreased the nuclear localization of HDAC3. (**A**) siRNA was used to knock down the expression of CBX4. The nuclear localization of HDAC3 was detected by Western blotting (**B**) and immunofluorescence (**C**). (**D**) Western blotting was used to measure Runx2 protein levels. (**E**–**H**) The mRNA levels of Osterix, OCN, OPN, and ALP were determined by PCR. (**I**) Bimolecular fluorescence complementation (BiFC) assay was performed to confirm the interaction between CBX4 and HDAC3 in vivo. *P < 0.05. **Figure S2.** miRNAs levels in ACP cells. (**A**) mimics of miRNAs were transfected into cells and Q-PCR was used to assay the levels of miRNAs. (**B**) inhibitors of miRNA were used to suppress miRNAs levels and miRNAs expression was analyzed by Q-PCR. *P < 0.05.

## Data Availability

All data in this study are available upon request.

## Abbreviations

ACP: Adamantinomatous craniopharyngioma
ALP: Alkaline phosphatase
Bmp2: Bone morphogenetic protein 2
BSA: Bovine serum albumin
CBX4: Chromobox 4
CP: Craniopharyngioma
CT: Computed tomography
HAT: Histone acetyltransferase
HDAC: Histone deacetylase
inh-181b: MiR-181b inhibitor
NC: Negative control/mimic controls
OCN: Osteocalcin
OPN: Osteopontin
Runx2: Runt-related transcription factor 2
SPCP: Squamous papillary craniopharyngioma
SUMO: Small ubiquitin-like modifier
