# Supplementary material for: CBX4-dependent regulation of HDAC3 nuclear translocation reduces Bmp2-induced osteoblastic differentiation and calcification in adamantinomatous craniopharyngioma
Source: Cell Commun Signal. 2022 Jan 3;20:3. doi: 10.1186/s12964-021-00797-w (PMC8722308; doi:10.1186/s12964-021-00797-w)
Supplement: Supplementary file 2 — Additional file 1: Figure S1. Knockdown of CBX4 decreased the nuclear localization of HDAC3. (A) siRNA was used to knock down the expression of CBX4. The nuclear localization of HDAC3 was detected by Western blotting (B) and immunofluorescence (C). (D) Western blotting was used to measure Runx2 protein levels. (E–H) The mRNA levels of Osterix, OCN, OPN, and ALP were determined by PCR. (I) Bimolecular fluorescence complementation (BiFC) assay was performed to confirm the interaction between CBX4 and HDAC3 in vivo. *P < 0.05. Figure S2. miRNAs levels in ACP cells. (A) mimics of miRNAs were transfected into cells and Q-PCR was used to assay the levels of miRNAs. (B) inhibitors of miRNA were used to suppress miRNAs levels and miRNAs expression was analyzed by Q-PCR. *P < 0.05. [file 12964_2021_797_MOESM2_ESM.pptx]

## Slide 1
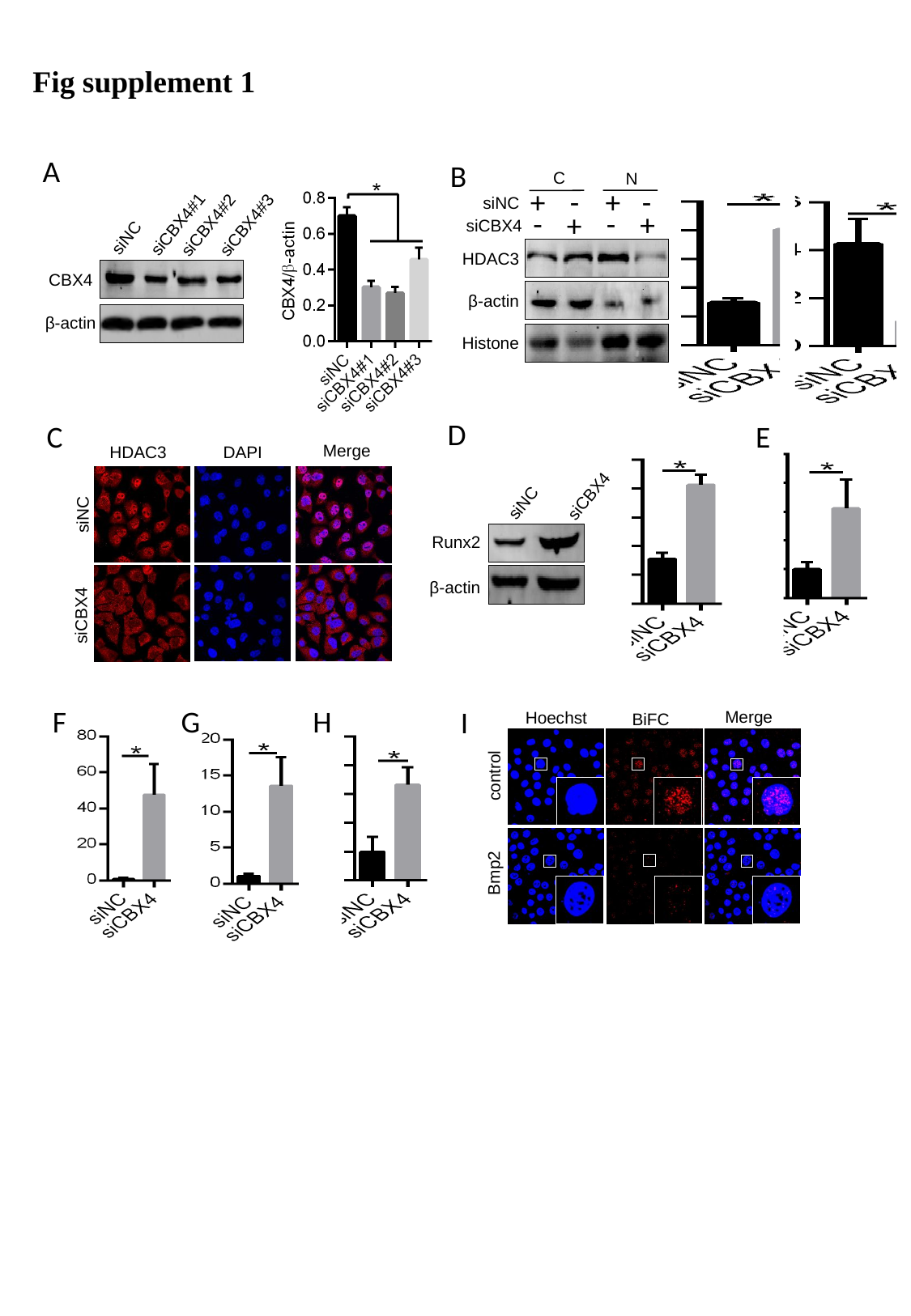

Fig supplement 1
A
C
N
-
-
+
+
siNC
-
-
+
+
siCBX4
HDAC3
β-actin
Histone
B
siCBX4#1
siCBX4#3
siCBX4#2
siNC
CBX4
β-actin
D
C
E
Merge
HDAC3
DAPI
siCBX4
siNC
siNC
Runx2
β-actin
siCBX4
F
G
H
I
Merge
Hoechst
BiFC
control
Bmp2

## Slide 2
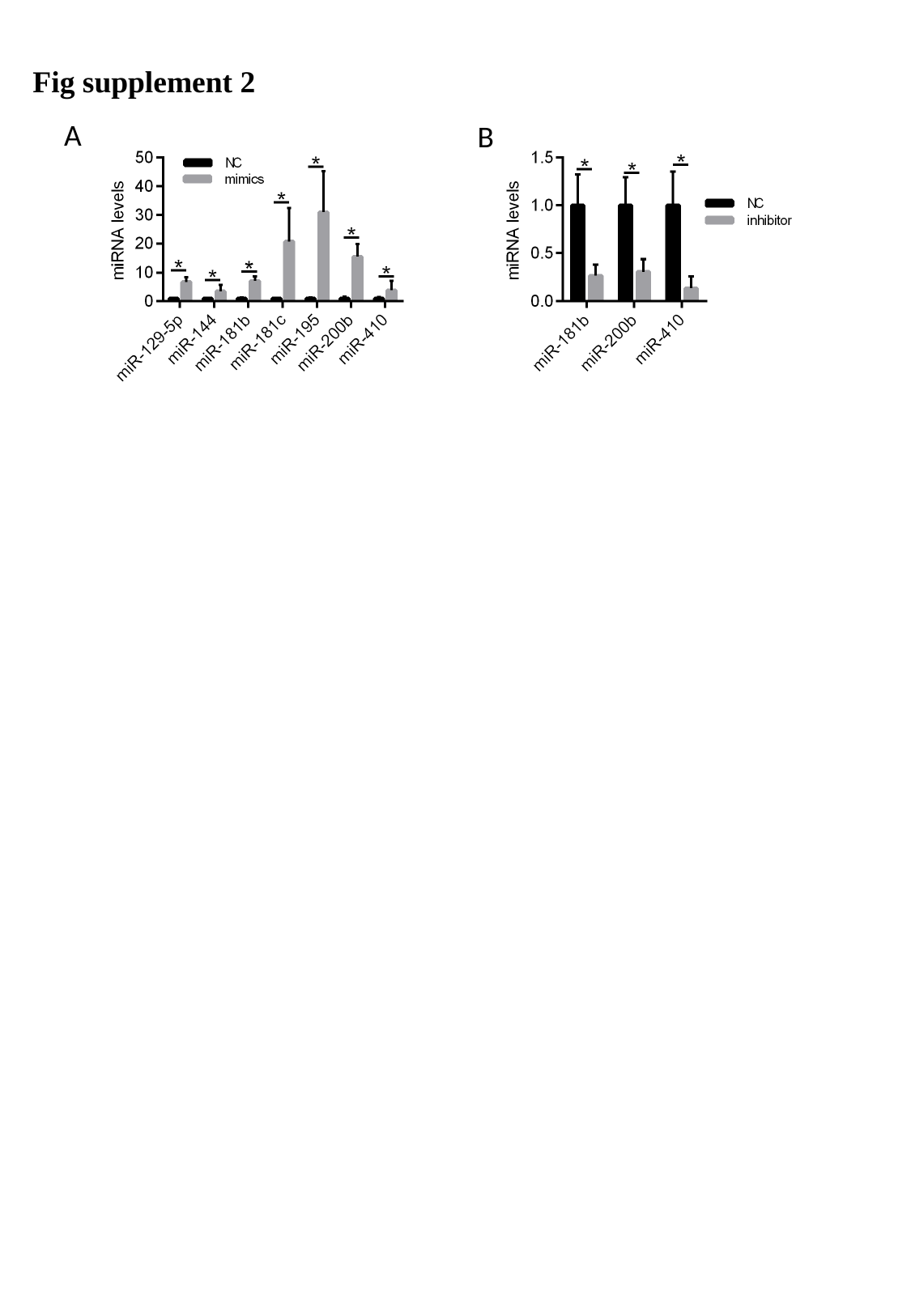

Fig supplement 2
A
B
